# PCR testing of traced contacts for SARS-CoV-2 in England, January to July 2021

**DOI:** 10.2807/1560-7917.ES.2023.28.44.2300019

**Published:** 2023-11-02

**Authors:** Toby Nonnenmacher, Niharika Dandamudi, Matthias Erwin Futschik, Sarah A Tunkel, Raghavendran Kulasegaran-Shylini, Nick Germanacos, Joanna Cole-Hamilton, Edward Blandford, Ashley Goddard, Joe Hillier, Stephen Finer, Susan Hopkins, Tom Fowler

**Affiliations:** 1 United Kingdom Health Security Agency, London, United Kingdom; 2 Faculty of Health, School of Biomedical Sciences, University of Plymouth, Plymouth, United Kingdom; 3 William Harvey Research Institute, Queen Mary University of London, London, United Kingdom

**Keywords:** NHS Test and Trace, PCR, contact tracing, SARS-CoV-2, COVID-19, contact testing

## Abstract

**Background:**

The NHS Test and Trace (NHSTT) programme was established in May 2020 in England to deliver SARS-CoV-2 testing and contact tracing in order to identify infected individuals and reduce COVID-19 spread. To further control transmission, people identified as contacts were asked to self-isolate for 10 days and test only if they became symptomatic. From March 2021, eligibility criteria for PCR testing expanded to include asymptomatic contacts of confirmed cases.

**Aim:**

To analyse testing patterns of contacts before and after the change in testing guidance in England to assess the impact on PCR testing behaviour with respect to symptom status and contact type.

**Methods:**

Testing and contact tracing data were extracted from the national data systems and linked. Subsequently, descriptive statistical analysis was applied to identify trends in testing behaviour.

**Results:**

Between 1 January and 31 July 2021, over 5 million contacts were identified and reached by contact tracers; 42.3% took a PCR test around the time they were traced. Overall positivity rate was 44.3% and consistently higher in symptomatic (60–70%) than asymptomatic (around 20%, March–June) contacts. The proportion of tests taken by asymptomatic contacts increased over time, especially after the change in testing guidance. No link was observed between uptake of PCR tests and vaccination coverage. Fully vaccinated contacts showed lower positivity (23.8%) than those with one dose (37.2%) or unvaccinated (51.0%).

**Conclusion:**

Almost 1 million asymptomatic contacts were tested for SARS-CoV-2, identifying 214,056 positive cases, demonstrating the value of offering PCR testing to this group.

**Key public health message**

**What did you want to address in this study?**

From May 2020 onwards, the NHS Test and Trace programme in England offered PCR testing to all individuals with COVID-19 symptoms. In March 2021, testing guidance changed, and testing was also offered to people with no symptoms who had been in contact with an infected person. We wanted to understand if this altered the way that people were choosing whether to test, and if we could detect new cases that might otherwise be missed.

**What have we learnt from this study?**

Through PCR testing of contacts, over 200,000 infected people with no symptoms were identified over the period of the study (January–July 2021). After the change in testing guidance, the proportion of contacts taking a PCR test increased steadily, peaking at nearly 50%, while the percentage of positive tests decreased. A positive test was more likely for people with symptoms, not vaccinated, or living in the same household.

**What are the implications of your findings for public health?**

PCR testing of contacts aided identification of infected individuals with or without symptoms. By continuing to monitor the way people tested after change testing guidance, it has been possible to assess the success of the new testing guidance, both in implementation and in detection of new cases that may not have presented clinically.

## Introduction

Since the start of the COVID-19 pandemic in early 2020, non-pharmaceutical interventions implemented across the world have included isolation of COVID-19 cases and their contacts to break the chains of virus transmission [1]. In England, the National Health Service Test and Trace (NHSTT) programme [2], established on 28 May 2020, aimed to ensure that anyone with symptoms of COVID-19 could be tested for severe acute respiratory syndrome coronavirus 2 (SARS-CoV-2) by PCR to determine if they were infected. Furthermore, NHSTT provided the Contact Tracing and Advisory Service (CTAS), which aimed to collect details of the contacts of individuals who tested positive for SARS-CoV-2, trace these contacts, and give advice on testing and self-isolation (referred to as quarantine in some countries). The functions of NHSTT were transferred to the newly established United Kingdom Health Security Agency (UKHSA) in October 2021 [3,4].

As knowledge developed during the pandemic, changes were made to the testing guidance for contacts [5,6]. From 30 March 2021, all contacts of confirmed COVID-19 cases became eligible to take a PCR test, whether or not they had symptoms. This was functionally implemented in two ways: (i) the scripts for contact tracers were amended to inform contacts that they should book themselves a PCR test, and (ii) the digital pathway for booking a PCR added an option for contacts of cases. Prior to this, with the exception of specific use cases, e.g. elective care testing, care home workers and outbreaks, PCR tests had only been available to the general public if they self-reported symptoms of COVID-19 or if they had tested positive for SARS-CoV-2 with a lateral flow device (LFD)/rapid antigen test.

In our study, we evaluate this change in testing guidance for contacts and explore its impact on subgroups by symptom status, vaccination status and type of contact among the population of contacts in England by analysing the period before and after the change was implemented i.e. from January to the end of July 2021. This analysis can help inform the future testing strategy in the case of occurrence of a new, more severe SARS-CoV-2 variant or a future pandemic.

## Methods

### Study setting

**Figure 1:**
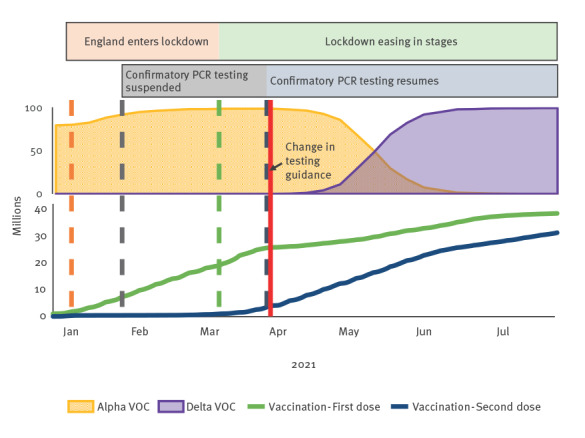
Timeline of restrictions, SARS-CoV-2 variants of concern and vaccinations, England, 1 January–31 July 2021

SARS-CoV-2: severe acute respiratory syndrome coronavirus 2; VOC: variant of concern.

At the beginning of the period analysed in our study in January 2021, the number of new COVID-19 cases reached a record level [7] and the Alpha variant (Phylogenetic Assignment of Named Global Outbreak (Pango) lineage designation B.1.1.7) was dominant in England (Figure 1). Vaccination was rolled out across the United Kingdom (UK), and England entered lockdown on 6 January 2021. As COVID-19 cases declined, lockdown measures eased while the Delta variant (Pango lineage designation B.1.617.2) became dominant around end of May 2021. 

COVID-19 vaccination programme started in England in December 2020 and used vaccines developed by BioNTech-Pfizer, Oxford-AstraZeneca and Moderna.

### Testing guidance

At the beginning of January 2021, PCR testing was restricted to individuals who had symptoms suggestive of COVID-19, and those who had received a positive LFD test result. This recommendation for confirmatory PCR testing following a positive LFD was suspended in January 2021 and resumed in March 2021 [8].

Contacts of individuals who returned a positive test result were only eligible for PCR testing if they displayed COVID-19 symptoms. These symptoms included any of the following: a new continuous cough, a high temperature or a loss of/change in sense of taste or smell. Following the change in testing guidance in March 2021, all contacts were eligible for PCR testing irrespective of symptom status.

From April, all individuals irrespective of their symptom status had free access to regular testing with LFDs as part of the Universal Testing Offer (UTO) [9]. This meant that, prior to the change in testing guidance for contacts in March 2021, contacts only had access to PCR testing if they showed COVID-19 symptoms. Contacts without symptoms could choose to test themselves with LFDs if they had access to them through workplace testing or a similar scheme before the UTO.

### Definition of index cases and contacts 

In England, all individuals with a positive result for SARS-CoV-2, from either an LFD or a PCR test, represented index cases and were notified to CTAS, which triggered a tracing process to identify contacts of the index case. A contact was defined as a person who (i) had contact with the index case within 1 m, (ii) spent more than 15 minutes within 2 m of the index case, (iii) travelled in the same car or other small vehicle with the index case or (vi) travelled in the same airplane with the index case up to 2 days before their positive test [10]. The definition used for symptomatic status of contacts was the presence of any COVID-19 symptoms that conferred eligibility for PCR testing. If an index case with a positive LFD result subsequently received a negative PCR result, the tracing process was stopped.

### Tracing of contacts

Newly detected index cases were reached by a contact tracer to collect personal details of their identifiable contacts, with 75% of the cases being reached within 24 h and 95% within 72 h [7]. These named contacts were then reached by phone or email, informed of their contact status and given instructions for testing (if indicated) and self-isolation. Sometimes a contact may already have been tested before they had been traced, for example, if they had symptoms and therefore sought testing on their own accord. The duration of self-isolation was 10 days, and this remained the case, as a legal requirement [11], throughout the period evaluated here. 

Additional statistics from NHSTT, such as the number of index cases or contacts reached, geographical distributions and time required for contacts to be reached, were published on weekly basis during the period of this evaluation and can be accessed at the United Kingdom (UK) government webpage [7].

### Data collection and linkage 

The results of PCR and LFD tests were stored in a central national database called National Pathology Exchange (NPEx). With different levels of completeness, NPEx also holds demographic data (age, sex, self-identified ethnicity and regions), data on vaccination status and self-reported symptoms. Contact tracing data are held within the CTAS database, including the date on which contacts were reached, and personal identifiable information. For contacts who were reached by contact tracers multiple times in a single week, only the first occasion that week was included. If a contact had been exposed to multiple index cases over 1 week, we assumed that they would take just one PCR test. 

The data in NPEx were linked with the data in the CTAS database by querying for an exact match on their personal identifiable information (surname, postcode and date of birth). Because contacts may have become symptomatic and were tested before being traced by CTAS, all matched PCR tests taken up to 5 days before or 8 days after being reached were included. This timeframe was based on the expectation that tests outside this window were not relevant to the contact event. In our study, the analysed population were those contacts who were successfully matched with a PCR test. 

### Statistical analysis

We performed a descriptive analysis, showing the absolute number and corresponding percentages of traced contacts, by calendar week of the date reached by the tracer, whether the contact had taken a PCR test, result of the first PCR test conducted 5 days before or 8 days after being reached, presence of symptoms, vaccination status and type of contacts (e.g. household, workplace or school contacts). The date used to assign a calendar week for each contact was the date the contact was first reached by contact tracers (even though the date of the PCR test may have been different). In this analysis, we used age, sex, ethnicity and geographical regions to define demographic groups. For comparison between the demographic composition of contacts and of the general population of England, national census data of 2020 (for sex, age and regions) and 2021 (for ethnicity) were used [12]. To assess whether certain groups were over- or underrepresented among contacts with respect to the general population, ratios of rates were calculated. These are defined as the ratio of the percentage for contacts to the corresponding percentage in the census data. A ratio larger than 1 indicates overrepresentation, while a ratio smaller than 1 indicates underrepresentation for any given demographic characteristic.

## Results

**Figure 2:**
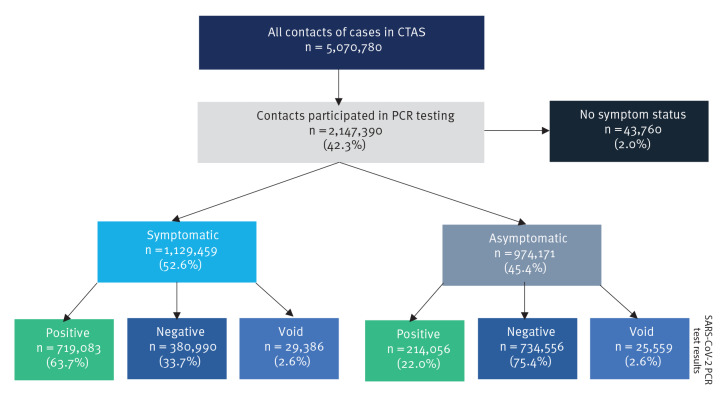
SARS-CoV-2 PCR tests results of traced contacts of confirmed COVID-19 cases by presence or absence of symptoms, England, 1 January–31 July 2021 (n = 5,070,780)

CTAS: Contact Tracing and Advisory Service; SARS-CoV-2: severe acute respiratory syndrome coronavirus 2.

**Figure 3:**
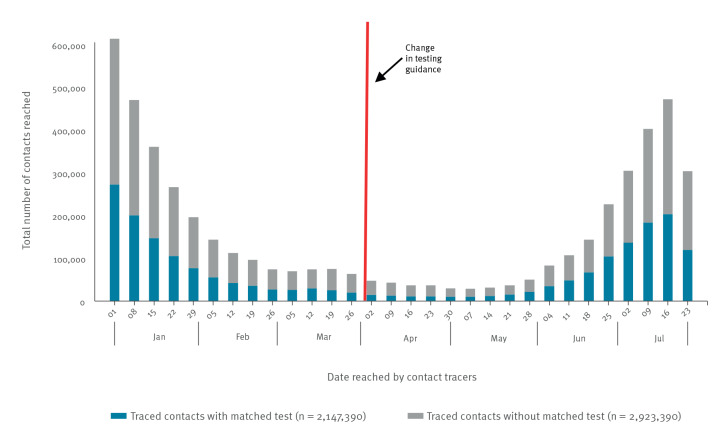
Number of contacts reached by contact tracers, with or without matched SARS-CoV-2 PCR tests, by week, England, 1 January–31 July 2021 (n = 5,070,780)

CTAS: Contact Tracing and Advisory Service; SARS-CoV-2: severe acute respiratory syndrome coronavirus 2.

For the period between 1 January to 31 July 2021 in England, there were 5,070,780 contacts of confirmed COVID-19 cases who were reached by contact tracers (Figure 2). Among the contacts, 2,147,390 (42.3%) had a matched PCR test between 5 days before and 8 days after the date of being reached by the contact tracer. Figure 3 shows the total number of contacts reached, split by whether they had a matched PCR result or not. The trend in the total number of contacts followed the estimated infection rate for England during the same time period [13]. For the analysed time period, the number of contacts was at its maximum at the start of 2021, and decreased to a low point in April and May 2021. An increase in the traced contacts was observed again from late May, closely following the increasing case numbers [7]. This coincided with the rise in dominance of the Delta variant and relaxation of social restrictions. There was no discernible change in the total number of contacts and percentage with matched PCR related to the time of the change in the testing guidance. In the following sections, we present the analysis of contacts with matched PCR.

‘Contacts participated in PCR testing’ refers to contacts of confirmed COVID-19 cases with a matched PCR test result from between 5 days prior to being traced to up to 8 days after being traced.

Voids were declared when test results could not be provided by the lab, which could be for any one of a variety of reasons ranging from sample leakage in transit to a technical void in lab processing.

The red line indicates the date when the change in testing guidance was implemented in England after which all contacts of cases were offered a PCR test regardless of symptom status.

Of the 2,147,390 contacts with a matched PCR, the majority were female (53.6%), white (80.8%), aged 21–40 years (35.3%), had symptoms (53.7%), and were from the North of England (32.4%) (Table 1). In comparison with the general population of England [12], the contacts consisted of a higher percentage of females, people of Asian ethnicities and people from the North of England, and they were younger. In contrast, individuals within the age groups 61–80 and over 80 years or with Black ethnicity were underrepresented among contacts. Of note, the number of contacts identified in the over 80 years age category was less than one tenth of what would be expected if the age distribution of contacts mirrored that of the general population.

**Table 1:** Characteristics of traced contacts who underwent PCR testing for SARS-CoV-2, England, January–31 July 2021 (n = 2,147,390), compared with estimates for the general population, England, mid-2020 and mid-2021^a^

Categories	Total n = 2,147,390	Percentage (%)		Ratio of rates
		Contacts	Census^a^	
Sex				
Female	1,150,161	53.6	50.5	1.06
Male	995,687	46.4	49.5	0.94
Prefer not to say	1,542	n/a		
Age (years)				
≤ 20	572,744	26.7	23.6	1.13
21–40	758,728	35.3	26.2	1.35
41–60	661,968	30.8	26.1	1.18
61–80	144,961	6.8	19.1	0.35
> 80	8,989	0.4	5.0	0.08
Missing data	0	n/a		
Ethnicity				
White	1,672,130	80.8	81.7	0.99
Asian	261,651	12.6	9.3	1.36
Black	58,202	2.8	4.0	0.70
Mixed	48,816	2.4	2.9	0.81
Other	27,665	1.3	2.1	0.64
Missing data	78,926	n/a		
Region				
London	332,309	15.5	15.9	0.97
Midlands and East	639,793	29.8	30.2	0.99
North	694,729	32.4	27.5	1.18
South East	303,427	14.1	16.3	0.87
South West	175,542	8.2	10.0	0.82
Other	1,590	0.1	2.1	0.04
Missing data	0	n/a		
SARS-CoV-2 PCR test				
Positive	951,629	45.5	n/a	
Negative	1,139,991	54.5		
Void	55,770	n/a		
Symptoms				
Asymptomatic	974,171	46.3	n/a	
Symptomatic	1,129,459	53.7		
Missing data	43,760	n/a		

n/a: not applicable; SARS-CoV-2: severe acute respiratory syndrome coronavirus 2.

^a^ Census data of 2020 for sex, age and regions and of 2021 for ethnicity general population in England were obtained from the Office for National Statistics websites [12]. The census is based on mid-year (30 June) estimates. The exact population estimates are 56,550,138 (2020) and 56,536,419 (2021).

The percentages were calculated over the sum of valid data, excluding the missing data or voids. Voids were declared when test results could not be provided by the lab, which could be for any one of a variety of reasons ranging from sample leakage in transit to a technical void in lab processing.

### Percentage of traced contacts by self-reported symptom status

**Figure 4:**
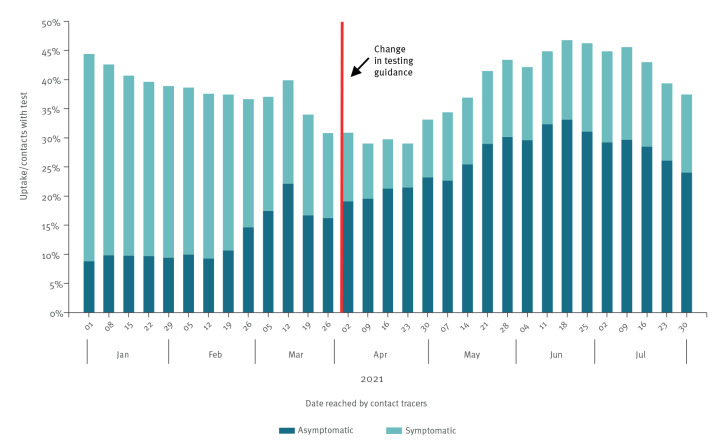
Percentage of traced contacts with a matched SARS-CoV-2 PCR test, with and without self-reported symptoms by week, England, 1 January–31 July 2021 (n = 2,103,630)

Overall, the weekly percentage of those contacts who had a matched PCR result fluctuated over time, decreasing from January 2021 and starting to increase again from late April (Figure 4). There was no step change observed in testing rate coinciding with the date of the change in testing guidance, but a steady and sustained rise was seen from April onwards. The highest level was recorded mid-June (46.8%) and the lowest (29.0%) in April. Over the whole study period, the percentage of contacts with matched PCR was 42.3%. The percentage of contacts without self-reported symptoms who took a PCR test increased over time: from 8.8% of total contacts at the start of the year to its highest level (33.1%) in the week of the 18 June, which coincided with the highest proportion of testing. While the proportion of asymptomatic contacts increased after the change in testing guidance, a trend towards higher proportion of asymptomatic contacts appears to precede the change. Approximately 20–30% of contacts who tested in January and early February were asymptomatic, rising to 40–50% in late February and March. After the change in testing guidance, 62–72% of contacts who tested were asymptomatic.

SARS-CoV-2: severe acute respiratory syndrome coronavirus 2.

The red line indicates the date when the new testing guidance was implemented in England, i.e. all contacts were offered a PCR test. Note that the y-axis has a reduced percentage range (0–50%).

### Positivity rate by symptom status

**Figure 5:**
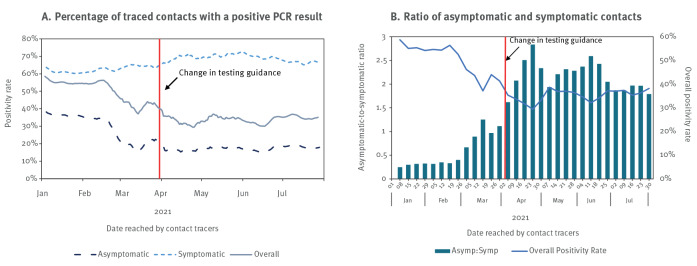
SARS-CoV-2 positivity rate of traced contacts by symptom status, England, 1 January–31 July 2021 (n = 2,103,630)

Figure 5A shows the positivity rate as a 7-day rolling average between January and July 2021, overall and separately for those with and without self-reported symptoms. We observed a high average overall positivity rate for traced contacts (44.3%). The rate among symptomatic contacts was consistently over 60% and considerably higher than those of asymptomatic contacts. There was more variation in the positivity rate in contacts without symptoms; it appeared stable until mid-February then decreased until mid-March, when there was a temporary upward trend before decreasing again to a stable level between 15% and 20% from April onwards. The overall positivity rate appears to correlate with the ratio of asymptomatic to symptomatic testers. As the proportion of asymptomatic testers rises, the positivity rate falls (Figure 5B).

SARS-CoV-2: severe acute respiratory syndrome coronavirus 2.

The red lines indicate the date when the new testing guidance was implemented in England.

### Positivity rate by vaccination and symptom status

Among all contacts who took a PCR test and who reported their symptom status (n = 2,103,630), 47.5% (n = 999,080) self-reported that they were not vaccinated at the time of taking their test, 12.5% (n = 262,592) that they had one dose and 19.5% (n = 409,974) that they had two doses of vaccination; 20.5% (n = 431,984) did not report their vaccination status. The contacts who had received two doses of COVID-19 vaccine showed lower overall positivity (23.8%) compared with those who only received one vaccine dose (37.2%) and those who were not vaccinated (51.0%) (Table 2). Also, contacts without symptoms had a lower positivity in all vaccination groups (no dose, 1 and 2 doses) compared to those with symptoms.

**Table 2:** SARS-CoV-2 positivity rate for traced contacts for whom self-reported vaccination and symptom status were available, England, 1 January–31 July 2021 (n = 1,631,891)

Number of vaccine doses	Total traced contacts with matched PCR (n)	Contacts with positive matched PCR (n)	Positivity rate (%)	Vaccination status (%)
**Contact with symptoms**				
None	581,011	380,656	65.5	73.7
1 dose	104,695	69,055	66.0	13.3
2 doses	103,026	60,499	58.7	13.1
**Total**	**788,732**	**510,210**	**64.7**	**100**
**Contact without symptoms**				
None	391,622	115,076	29.4	46.4
1 dose	152,431	26,709	17.5	18.1
2 doses	299,106	35,402	11.8	35.5
**Total**	**843,159**	**177,187**	**21.0**	**100**
**All contacts**				
None	972,633	495,732	51.0	59.6
1 dose	257,126	95,764	37.2	15.8
2 doses	402,132	95,901	23.8	24.6
**Total**	**1,631,891**	**687,397**	**42.1**	**100**

SARS-CoV-2: severe acute respiratory syndrome coronavirus 2.

COVID-19 vaccination programme started in England in December 2020 and used vaccines developed by BioNTech-Pfizer, Oxford-AstraZeneca and Moderna. Percentage for vaccination status was calculated based on sum of valid self-reports. Contacts with available self-reported vaccination and symptom status but with a void PCR test were excluded (n = 39,755).

### Percentage of PCR tests and positivity rate by type of contact

**Figure 6:**
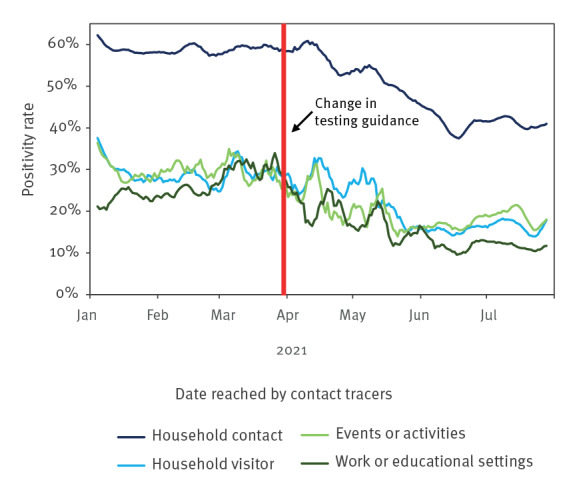
SARS-CoV-2 positivity of traced contacts by contact type, as a 7-day rolling average, England, 1 January–31 July 2021 (n = 2,147,390)

Of the contacts who were tested by PCR, the vast majority were household contacts. After the change in testing guidance, the proportion of other types of contacts including household visitors, contacts at events or activities, and contacts through work or educational settings began to increase. This shift in the distribution could be due to changes in other policies such as the physical re-opening of schools in March 2021, and the gradual easing of restrictions (re-opening of non-essential services and mixing of households) from late March 2021, after which index cases may have had more contact with people outside of the household. When assessing the positivity rate by contact type, we observed that it differed between contact type and changed over the study period (Figure 6). The positivity rate was highest in household contacts at ca 60% until the change in testing guidance, after which the rate began to decrease, stabilising in late June at around 40%. The mean positivity rate for household contacts over the whole period was 52%. The positivity rate for other contact types was around 30% before the change of testing guidance, when it began to fall to around 10–20% in late May. Contacts from work or educational settings had the lowest overall positivity rate at ca 20%, with household visitor and activity or event overall positivity rates of around 24%.

SARS-CoV-2: severe acute respiratory syndrome coronavirus 2.

## Discussion

In November 2020, the European Centre for Disease Prevention and Control updated its contact tracing recommendations, advising countries to include testing of asymptomatic contacts as part of their national COVID-19 response [14]. In England, the main aim of the tracing activities of NHSTT was to reach every individual who tested positive for SARS-CoV-2, collect details of their contacts, trace these contacts and give advice on appropriate COVID-19 testing and the legal requirements for self-isolation. Over the whole lifespan of CTAS, more than 95% of identified contacts were reached within 24 h of their details being provided by index cases [7]. 

Estimates of the proportion of SARS-CoV-2-positive individuals who are asymptomatic vary, though it is thought that it could be between a fifth and a third of cases [15-18]. Being asymptomatic at the time of testing positive does not rule out the development of symptoms at a later stage of infection, and many who are defined as ‘asymptomatic’ may in fact be pre-symptomatic. For the original SARS-CoV-2 strain, transmission was more likely during the pre-symptomatic stage of infection [19]. Given that it is challenging to distinguish between asymptomatic and pre-symptomatic, and that transmission can occur both with and without symptoms, identifying cases at the earliest opportunity was considered important in order to trace contacts and initiate public health measures such as self-isolation.

Analysis of the COVID-19 Rapid Survey of Adherence to Interventions and Responses (CORSAIR) found that adherence to self-isolation was significantly increased if affected individuals attributed current symptoms to COVID-19 [20]. This indicated that individuals were more likely to comply with self-isolation if they knew they had COVID-19, confirmed through testing. With both the intention to reduce asymptomatic or pre-symptomatic transmission, as well as to increase compliance with self-isolation, the change in testing guidance was implemented. Additional reasons for the new guidance were: (i) not all individuals could recognise the main symptoms of COVID-19 [20], (ii) delays in testing strongly influenced the efficacy of contact tracing [21], (iii) the main symptoms used to determine eligibility for PCR testing may not present in all cases [22] and (iv) the majority of contacts were household contacts who were at the highest risk of infection [23].

Through the course of the study, the number of contacts traced and tested varied. It was as high as 613,000 contacts on 1 January 2021, but dropped with a national lockdown in place from the 6 January 2021 onwards and with increasing vaccination uptake of the population. The number of contacts increased again as lockdown measures were eased and the Delta variant became dominant around the end of May 2021.

From 1 January to 31 July 2021, 42.3% of contacts reached by CTAS were matched to a PCR test around the time they were reached. All contacts had access to use of LFD tests from April 2021 when the UTO commenced which may have had an impact on uptake of PCR testing. There could be many other factors contributing to rate of uptake, including unwillingness to be tested, lack of awareness of the option to test and issues with the data matching because of missing personal details. The notable impact of the guidance change was the increase in rate of testing of contacts who were asymptomatic, as these were the people who were previously ineligible to seek a PCR. However, it had been observed from real-time data monitoring that a substantial proportion of contacts who had PCR tests prior to the guidance change did not have symptoms, and it was therefore proposed that new guidance would be popular and easy to implement.

A qualitative study [24] found that when symptoms were mild, people did not necessarily attribute them to COVID-19 and found it more difficult to decide what to do, with most saying they would ‘wait and see’ if the symptoms developed further before taking a test. Others have also found that, in the UK, those with only one symptom were less likely to seek testing than those with multiple symptoms and that, of those who wanted to test but did not, the most common reason was not knowing where or how to get a test [25]. Nevertheless, the percentage of contacts who took a PCR test was approximately double that of the rather low percentage (22.2%) of individuals who requested a test after symptoms developed, as determined by CORSAIR for late January 2021 [20]. This suggests that being reached by NHSTT greatly increased the likelihood of taking a test.

Analysis of the demographic characteristics showed that contacts with a matched PCR tended to be younger (< 60 years), while those over 80 years showed the strongest underrepresentation compared with the general population. One reason could be that older individuals might have avoided taking a test. However, the CORSAIR study did not show that age was a factor in uptake of tests [20]. Thus, the notable lack of matched PCR tests for the over 80s appears to be more likely an indication that the government guidance for shielding of the more vulnerable group of elderly people was effective in England, at least for the period of study [26]. Furthermore, females represented a higher proportion of matched PCR tests compared with males and compared with the general population, a finding consistent with results of the CORSAIR study showing that symptomatic females are significantly more likely to take a test than symptomatic males. We also found differences between ethnic groups, as contacts who reported Asian ethnicity were overrepresented, while those belonging to Black ethnicity were underrepresented. However, socioeconomic factors, which we did not consider in our analysis, might play a confounding role here and warrant further investigation. 

The existence of PCR testing data for asymptomatic traced contacts before the implementation date of the change in testing guidance is reflective of individuals who conducted a PCR test and marked their status as asymptomatic when prompted, despite guidance at the time recommending PCR testing only for symptomatic individuals. Contrary to our expectations, we did not detect a step change in the percentage of traced contacts who took a PCR test following the implementation of the new testing guidance. However, the proportion of contacts taking a PCR who reported themselves as being asymptomatic increased over the study period from 20% to up to 70%. Interestingly, the increase in the proportion of asymptomatic contacts preceded the change in guidance and 52% of the contacts with matched tests were asymptomatic in the week before the change. One possible explanation for this increase is that this cohort of asymptomatic contacts could have been testing regularly with LFDs and simply chose to take PCR tests, given PCR is more sensitive and can detect infection earlier in the infection period than LFDs. There were various national level targeted testing programmes, such as residents and staff of care homes [27,28] and people accessing elective healthcare [29], that were in place in England and asymptomatic contacts could have been tested outside the COVID-19 tracing service.

Of all the traced contacts who took PCR tests, the most frequent contact type was an individual belonging to the same household as the index case; this type also showed the highest positivity rate across the study period. This finding is in line with evidence from other studies that demonstrated a high secondary attack rate within households, i.e. contacts living in a household were more likely to test positive [21,30]. Despite the easing of restrictions, positivity of contacts dropped across the different types of contacts (household, household visitor, work, educational settings and events [31]). This drop in contact positivity across different contact types was likely to be a direct result of increased vaccination coverage, as positivity rate over the whole period was twice as high in unvaccinated contacts than doubly vaccinated contacts. This finding is consistent with the REACT-13 study that assessed PCR test data in England between 20 May and 12 July 2021 and showed that SARS-CoV-2 infections were three times less frequent in people who were fully vaccinated with two doses compared with unvaccinated people, and after adjusting for age and other factors, vaccine effectiveness against symptomatic infection was estimated at around 50–60% [32]. However, this protective effect might not be long-lasting; a study from 2022 using contact tracing data from England, which included adult contacts in the same time period as the evaluation presented here, reported that vaccination only lowers transmission rates in the 12 weeks following vaccination [33].

There are several limitations to our study. Firstly, the matching process was dependent on the quality of the data provided. If personal identifying information was not provided or was provided incorrectly, matching an individual reached by NHSTT who had taken a PCR test was not possible. It should be noted that demographic data, symptom status and vaccination status were self-reported by contacts. Secondly, transmission networks are highly complex. A contact may have had multiple opportunities to be exposed to the virus and the case-contact pairing identified in the data may not be the actual transmission route for those contacts who test positive. Thirdly, some individuals tested in the days before being reached, entirely independently of the contact event in question. These individuals’ results would have been included in the analysis. Deduplication was carried out for multiple instances of the same individual being reached by NHSTT within 7 days. Fourthly, for traced contacts who did not test, we do not have demographic data which could be used to identify underlying factors. Lastly, socioeconomic status, which was significantly associated with the intention to share contact details in the CORSAIR study [20], was not included in the study data. Further analysis would be required to understand its relationship with testing behaviours of contacts.

Furthermore, our study assessed the impact of PCR testing only, and not LFD testing. Testing with LFDs may be a feasible alternative if PCR testing is constrained or otherwise not available [34]. Regular testing using LFDs can be an efficient and less costly way to identify and isolate infectious individuals, preventing further transmission of infection [35]. Indeed, LFDs were robust in detecting SARS-CoV-2 infections throughout vaccine roll-outs and across different viral variants, although the lower performance for asymptomatic individuals might warrant further consideration [35].

## Conclusions

Our findings confirmed the value of offering PCR testing to all named contacts of positive index cases of COVID-19 regardless of their symptom status in England. Our analysis demonstrates that an estimated 1 million asymptomatic individuals who were potentially excluded before the testing guidance change became eligible for testing. Through this change in testing guidance, over 200,000 asymptomatic contacts tested positive and were required to self-isolate, breaking chains of COVID-19 transmission. This study demonstrates that despite the considerable resource implications of contact tracing in pandemic or major epidemic situations, it is possible to successfully implement this public health intervention. Our work also suggests that the effectiveness of testing of contacts via contact tracing in a future pandemic scenario may depend on the characteristics of the pathogen, which would need to exhibit substantial asymptomatic transmission, and should be designed to adapt flexibly in response to mutations of the pathogen. 
